# Cerebral oxygenation response to postural change using continuous near-infrared spectroscopy (NIRS) and its association with falls in older adults

**DOI:** 10.1007/s41999-026-01538-3

**Published:** 2026-06-29

**Authors:** Liping Wang, Eveline P. van Poelgeest, Marjolein Klop, Jurgen A. H. R. Claassen, Alfons G. Hoekstra, Nathalie van der Velde

**Affiliations:** 1 Department of Internal Medicine/Geriatrics, Amsterdam University Medical Centers, Meibergdreef 9, Amsterdam, The Netherlands; 2 Amsterdam Public Health, Aging and Later Life, Amsterdam, The Netherlands; 3 Department of Geriatrics, Radboud University Medical Center, Nijmegen, The Netherlands; 4 Computational Science Lab, Informatics Institute, Faculty of Science, University of Amsterdam, Amsterdam, The Netherlands; 5 Department of Cardiovascular Sciences, University of Leicester, Leicester, UK

**Keywords:** Cerebral oxygenation, Falls, Older adults, Near-infrared spectroscopy (NIRS), Orthostatic hypotension

## Abstract

**Background:**

Impaired cerebral perfusion during postural changes may contribute to fall risk in older adults. This study used near-infrared spectroscopy (NIRS) as a proxy for cerebral blood flow to investigate the association between cerebral oxygenation responses during postural changes and falls in older adults.

**Methods:**

We analyzed parameters of oxygenated hemoglobin (OxyHb), deoxygenated hemoglobin (DeoxyHb), total hemoglobin (tHb), and tissue saturation index (TSI) during supine-to-stand and sit-to-stand maneuvers in the PROHEALTH cohort of older adults (*n* = 30; 23% with positive falls history; mean age 74 ± 7 years). Associations with falls were assessed using logistic regression.

**Results:**

Significant associations with falls history were observed during the supine-to-stand maneuver, despite no significant differences in orthostatic cardiovascular responses. After adjusting for age and sex, lower OxyHb nadir-to-overshoot (OR = 5.20, 95% CI 1.06–25.57), lower TSI overshoot (OR = 1.86, 95% CI 1.04–3.33), and lower TSI initial recovery amplitude (OR = 1.86, 95% CI 1.04–3.34), all within the first minute of standing, were significantly associated with falls history. In addition, lower TSI steady-state values at 60–120 s after standing (OR = 1.56, 95% CI 1.01–2.42) also showed a significant association. No significant associations were observed for any of the parameters during the sit-to-stand maneuver.

**Conclusions:**

Impaired OxyHb and TSI responses, particularly within the first minute after standing and reduced TSI during sustained impaired recovery (60–120 s), were associated with falls in older adults. Supine-to-stand testing outperformed sit-to-stand testing in identifying cerebral oxygenation impairments. These findings highlight the clinical potential of NIRS as a practical tool for identifying orthostatic cerebral hypoperfusion-related fall risk. Further validation in larger, prospective cohorts is needed.

**Supplementary Information:**

The online version contains supplementary material available at https://doi.org/10.1007/s41999-026-01538-3.

## Key summary points

**Aim:**

To investigate the association between cerebral oxygenation responses during postural changes and falls in older adults, using near-infrared spectroscopy (NIRS) as a proxy for cerebral blood flow.

**Findings:**

Both early and sustained impairments in NIRS-measured cerebral oxygenation during standing were associated with falls in older adults. Supine-to-stand testing more effectively identified these impairments than sit-to-stand, with OxyHb nadir-to-overshoot and TSI parameters (overshoot, initial recovery, and steady-state) emerging as key markers.

**Message:**

NIRS-based monitoring during a standardized supine-to-stand test is promising as a potential clinical tool to identify older adults with orthostatic cerebral hypoperfusion-related fall risk.

**Supplementary Information:**

The online version contains supplementary material available at https://doi.org/10.1007/s41999-026-01538-3.

## Introduction

Falls in older adults are a major public health concern, leading to significant morbidity, mortality, and healthcare burden [1]. Impaired systemic blood pressure (BP) regulation upon standing is consistently linked to an increased risk of falls in older adults [2, 3]. Physiologically, cerebral autoregulation maintains stable cerebral perfusion despite fluctuations in systemic BP [4, 5]. However, for the relatively fast changes in BP that occur during postural transitions, autoregulation is unable to fully compensate for reductions or fluctuations in cerebral blood flow (CBF), resulting in transient cerebral hypoperfusion [5]. This hypoperfusion can lead to orthostatic symptoms, such as dizziness, light-headedness, and even syncope [5, 6].

Near-infrared spectroscopy (NIRS) is an increasingly utilized, continuous, non-invasive technique to assess cerebral oxygenation real-time (with every heartbeat), serving as a proxy for cerebral perfusion. The technique is suitable for assessment of cerebral oxygenation impairments during postural transitions [7–9], and is increasingly being used in tilt-table testing or in active stand testing [7, 10–14]. It correlates well with MRI and transcranial Doppler measures, demonstrating reliable tracking of cerebral perfusion, especially during supine-to-stand maneuvers [7, 10, 15–18]. Its portability, ease of use, and cost-effectiveness enhance clinical applicability [8, 19]. Importantly, the technique provides insights into central perfusion not captured by peripheral BP measures and shows promise in evaluating orthostatic intolerance, OH, and syncope [6, 9, 10, 14, 20–24]. Consistent with this, studies have demonstrated that reductions in cerebral oxygenation have been observed in patients with syncope even before peripheral hemodynamic changes are apparent, highlighting the sensitivity of NIRS in detecting early cerebral hypoperfusion [11, 13, 14, 23].

Although emerging evidence has also linked impaired cerebral oxygenation recovery to fall-related features such as postural instability [25], slower gait speed [8] and orthostatic hypotension (OH)/orthostatic intolerance [22, 23, 26], the associations between NIRS-measured oxygenation responses and fall risk in older adults remain largely unexplored. The recent studies suggest that early-phase NIRS responses, particularly within the first 30–40 s after standing, may have diagnostic value in identifying individuals with syncope, frailty, or multimorbidity [27, 28].

Given this knowledge gap and the potential clinical relevance of cerebral oxygenation monitoring beyond conventional orthostatic cardiovascular measures, this study aims to investigate the relationship between NIRS-based cerebral oxygenation responses during standing and falls in a cohort of older adults (using both supine-to-stand and sit-to-stand maneuvers), and to identify key NIRS parameters associated with falls. We hypothesized that impaired cerebral oxygenation responses upon standing, especially in the early phase after standing, would be associated with falls in older adults.

## Methods

### Data sources

We analyzed the dataset from a clinical study conducted at the Department of Geriatrics, Radboud University Medical Center (UMC), Nijmegen, the Netherlands: the PROHEALTH study, in which cerebral oxygenation was continuously measured by NIRS during postural changes, including 30 participants aged ≥ 65 years (healthy volunteers and > 30% from the geriatric outpatient clinic) [12]. Details on the study protocol and inclusion criteria have been published previously [12]. All participants provided written informed consent. The PROHEALTH study was deemed exempt from formal ethical review by the local ethics committee (CMO Radboud UMC), as it was concluded that it did not fall under the Medical Research Involving Human Subjects Act (WMO). The study was conducted in accordance with the Declaration of Helsinki.

### Participants characteristics

Baseline information included age, sex, height, weight, body mass index (BMI), comorbidity, and medication use (e.g., antihypertensives). Cognitive performance was assessed using the Montreal Cognitive Assessment (MoCA) in the PROHEALTH cohort [29]. BP was continuously measured using a Finapres device. Orthostatic intolerance symptoms were assessed using an OH questionnaire, including items such as “Do you ever experience light-headedness, dizziness, or the feeling of fainting when standing up?” and “Do you ever feel fatigue when or immediately after standing up?”. Depression and cardiovascular disease (including heart failure, myocardial infarction, other heart disease, stroke, cerebral hemorrhage, or cerebral infarction) were assessed using The Older Persons and Informal Caregiver Survey-Short Form (TOPICS-SF) comorbidity questionnaire completed by participants [30].

### Falls outcomes

In the PROHEALTH cohort, falls were assessed based on self-reported fall history at baseline, defined as positive if participants reported ≥ 1 fall in the previous year.

### Orthostatic cerebral oxygenation assessments

**Fig. 1:**
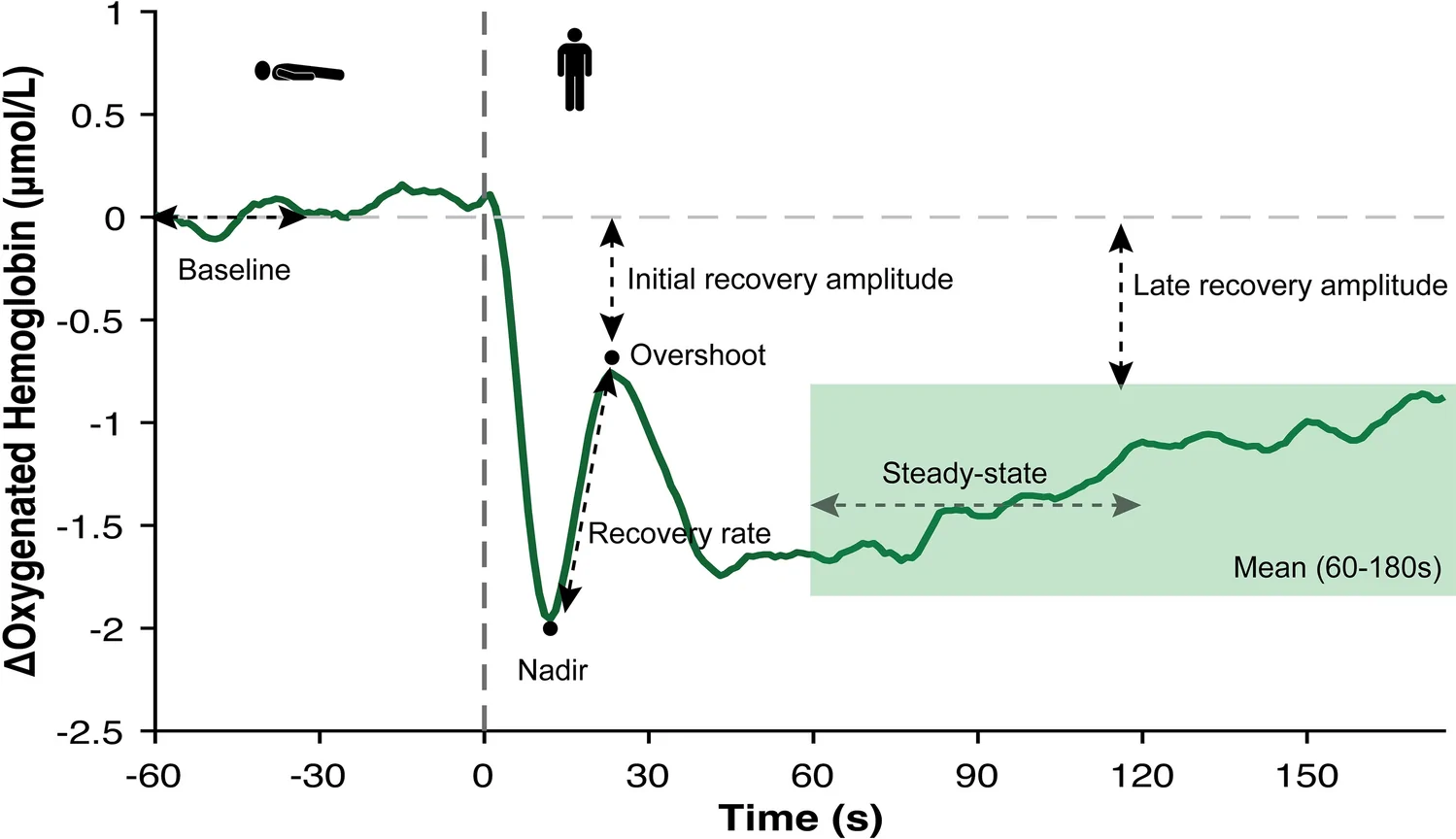
Visualization of variables used in the analyses. Depicted are the parameters for oxygenated hemoglobin. The same parameters were computed for total hemoglobin, tissue saturation index, and deoxygenated hemoglobin. Adapted from “*Determinants of orthostatic cerebral oxygenation assessed using near-infrared spectroscopy”* by Mol et al. (2022, 238, p3) and from *“Increased multimorbidity is associated with impaired cerebral and peripheral hemodynamic stabilization during active standing”* by Pérez-Denia et al. (2022, 70, p1976). For additional information (among other definitions) see Box 1

Cerebral oxygenation was continuously monitored during active stand tests using a NIRS device with bilateral forehead sensors (PortaLite MkII, Artinis Medical Systems, Elst, the Netherlands). The NIRS device was used to collect changes in the concentrations of oxygenated hemoglobin (OxyHb), deoxygenated hemoglobin (DeoxyHb), and total hemoglobin (tHb), relative to the baseline supine or sitting value. All NIRS-derived parameters were analyzed as relative changes from a standardized baseline, defined as the average value from 60 to 30 s before standing [8, 21]. tHb represents the sum of the changes in OxyHb and DeoxyHb and reflects cerebral blood volume dynamics [31]. The tissue saturation index (TSI), defined as the percentage of oxygenated to total hemoglobin and automatically derived from NIRS absolute concentration values through spatially resolved spectroscopy computation [7, 10, 32], reflects the dynamic balance between oxygen supply and consumption [33]. NIRS variables of interest and their measurement timings were defined based on previously published studies in the field (Box 1 and Fig. 1).

### Orthostatic maneuvers

In the PROHEALTH study, participants performed repeated supine-to-stand and sit-to-stand maneuvers separately, with a minimum of 5 min of rest followed by 3 min of standing [12]. For our analysis, we focused on the first supine-to-stand and sit-to-stand maneuver, as it ensured adequate rest before standing and was not potentially influenced by prior postural transitions.

**Box 1:** Definitions, units, description of variables used in the analyses (for visual representation see Fig. 1)

Variable	Description
Oxygenated Hemoglobin (OxyHb; μmol/L)	Changes in oxygenated hemoglobin concentrations relative to the baseline supine/sitting value, measured via NIRS [7, 10, 12]
Deoxygenated Hemoglobin (DeoxyHb; μmol/L)	Changes in deoxygenated hemoglobin concentrations relative to the baseline supine/sitting value, measured via NIRS [10, 12]
Total Hemoglobin (tHb; μmol/L)	The sum of the changes in OxyHb and DeoxyHb; reflects cerebral blood volume dynamics [31]
Tissue saturation index (TSI; in %)	Oxygenated hemoglobin as a percentage of total hemoglobin (automatically derived from NIRS absolute concentration values through spatially resolved spectroscopy computation [7, 10, 12, 32]
Orthostatic cerebral oxygenation response	
*Oxygenated Hemoglobin (OxyHb; μmol/L)*	
OxyHb* baseline	Mean value -30 to -60 s from standing [8, 21]
OxyHb* nadir	Largest drop after standing (in first 30 s): initial drop amplitude [27, 34]
OxyHb* overshoot	First peak found between nadir time + 2 s and 60 s after standing: temporary excess amplitude after initial drop [27, 34]
Delta * OxyHb	Nadir minus baseline [27, 34]
OxyHb* nadir-to-overshoot	Overshoot minus nadir [27, 34]
OxyHb* recovery rate (μmol/L/s)	(Overshoot minus nadir)/ (Overshoot time minus nadir time): largest difference between consecutive points divided by the time between points (within the time interval between the nadir and overshoot) [27, 34]
OxyHb* steady-state	Mean value 60-120 s after standing [27, 34]
OxyHb* initial recovery amplitude	Overshoot minus baseline: value of the first peak after the initial drop minus baseline [10]
OxyHb* late recovery amplitude	Mean in the interval between 60-180 s after standing up minus baseline [10]
OxyHb* at 10 s, 20 s, 30 s, 40 s	Value at 10 s, 20 s, 30 s, and 40 s after standing up, relative to the supine/sitting baseline [27]
*Deoxygenated Hemoglobin (DeoxyHb; μmol/L)*	
DeoxyHb baseline	Mean value -30 to -60 s from standing [8, 21]
DeoxyHb peak	Largest peak after standing (in first 30 s): initial increase amplitude [27, 34]
Delta DeoxyHb	Peak minus Baseline [27, 34]
DeoxyHb recovery rate (μmol/L/s)	Largest difference between consecutive points divided by the time between points (within the time interval between the nadir and peak) [27, 34]
DeoxyHb steady-state	Mean value 60-120 s after standing [27, 34]
DeoxyHb initial recovery amplitude	Value of the first drop after the initial increase minus baseline [10]
DeoxyHb late recovery amplitude	Mean in the interval between 60 and 180 s after standing up minus baseline [10]
DeoxyHb at 10 s, 20 s, 30 s, 40 s	Value at 10 s, 20 s, 30 s, and 40 s after standing up, relative to the supine/sitting baseline [27]
Orthostatic cardiovascular response	
Initial orthostatic hypotension	A systolic blood pressure (SBP) drop ≥ 40 mmHg and/or a diastolic blood pressure (DBP) drop ≥ 20 mmHg within 15 s after standing [35–37]. BP was continuously measured using Finapres device. Symptoms were not taken into account
Sustained orthostatic hypotension	A reduction in SBP of ≥ 20 mmHg or DBP of ≥ 10 mmHg, maintained across all timepoints between 60 and 120 s after standing [38]. BP was continuously measured using Finapres device
Classical orthostatic hypotension	A sustained SBP drop of ≥ 20 mmHg and/or a DBP drop ≥ 10 mmHg upon standing, defined as exceeding these thresholds at all following time points: 1, 2, and 3 min [39, 40]. BP was continuously measured using Finapres device

*CBF* cerebral blood flow, *DBP* diastolic blood pressure, *DeoxyHb* deoxygenated hemoglobin, *OxyHb* oxygenated hemoglobin, *s* second, *SBP* systolic blood pressure.

*The same parameters were computed for total hemoglobin (tHb; μmol/L) and tissue saturation index (TSI; in %).

### Statistical analysis

Baseline characteristics of the participants were summarized using descriptive statistics. The normality test (Shapiro–Wilk test) was performed to check if data followed a normal distribution. For variables with a normal distribution, mean values and standard deviations (SDs) were reported, while medians and interquartile ranges (IQRs) were used for variables that were not normally distributed. Categorical variables were presented as frequencies and percentages. To compare differences between participants with and without a history of falls, appropriate statistical tests were applied: the Chi-squared test or Fisher’s exact test for categorical variables, and the independent *t* test for normally distributed continuous variables or the Mann–Whitney *U* test for non-normally distributed continuous variables.

Cerebral oxygenation response parameters that showed significant group differences between participants with and without a history of falls were subsequently entered into logistic regression models to explore their associations with falls [41]. Binary logistic regression analysis was conducted to estimate odds ratios (ORs) with 95% confidence intervals (CIs) to evaluate the association between orthostatic cerebral oxygenation responses and falls. To aid interpretation, selected parameters were multiplied by -1 prior to analysis, so that OR > 1 consistently reflects higher fall risk associated with lower original parameter values. Owing to the relatively small sample size, models were adjusted only for age and sex as covariates [36].

The NIRS data were processed using MATLAB R2022a (MathWorks Inc., Natick, Massachusetts, USA). NIRS signals were resampled at 10 Hz and smoothed with a 5 s moving average filter to reduce artifacts. When both left and right signals were available and of sufficient quality, they were averaged; otherwise, the signal from the better-quality side was used. Missing data or signals deemed incomplete or excessively noisy upon visual inspection were excluded from further analysis, in line with approaches used by other research groups [8, 12, 42]. Specific NIRS variables (e.g., change and recovery of OxyHb, DeoxyHb, tHb, or TSI) were identified and extracted using custom-written scripts in MATLAB.

Statistical analyses were performed using MATLAB, RStudio (2024.12.1, R version 4.4.3), and SPSS (version 29.0, IBM Corp, NY). A *p* value of < 0.05 was set as the threshold for statistical significance, and trends were considered at a *p* value of < 0.1 [25, 43].

## Results

The mean age of participants in the PROHEALTH cohort was 74 ± 7 years old, and 37% were female. Twenty-three percent reported a history of falls in the past year. Table 1 presents the characteristics of participants with and without a history of falls. No significant differences in orthostatic cardiovascular responses, including the presence of OH, were observed between the two groups. For the NIRS analyses, data from one participant in the supine-to-stand maneuver and one participant in the sit-to-stand maneuver were excluded from analysis due to missing signals, flatline signals, noise, or poor waveform quality.

**Table 1:** Participant characteristics from the PROHEALTH cohort, comparing those with and without a history of falls

Characteristics	All (*n* = 30)	Fall previous year (*n* = 7; 23%)	No fall previous year (*n* = 23; 77%)	*P* value*
Age (years; mean ± SD)	74 ± 7	77 ± 6	73 ± 7	0.278
Female sex (*n*; %)	11 (37)	3 (43)	8 (35)	1.000
BMI (kg/m^2^; mean ± SD)	24 ± 3	25 ± 4	23 ± 3	0.266
MoCA score (median, IQR)	26 (24–28)	26 (24–27)	26 (24–28)	0.321
Initial OH (*n*; %)	9 (30)	1 (14)	8 (35)	0.393
Sustained OH (*n*; %)	10 (33)	3 (43)	7 (30)	0.657
Classical OH (*n*; %)	6 (20)	2 (29)	4 (17)	0.603
Orthostatic intolerance during measurements (*n*; %)	5 (17)	2 (29)	3 (13)	0.565
Cardiovascular disease (*n*; %)	4 (13)	2 (29)	2 (9)	0.225
Depression (*n*; %)	3 (10)	2 (29)	1 (4)	0.128
Antihypertensive drug use (*n*; %)	7 (23)	1 (14)	6 (26)	1.000
Antidepressant use (*n*; %)	2 (7)	1 (14)	1 (4)	0.418
Statin use (*n*; %)	3 (10)	1 (14)	2 (9)	1.000

*BMI* body mass index, *IQR* interquartile range, *MoCA* montreal cognitive assessment (maximum score = 30), *OH* orthostatic hypotension, *SD* standard deviation

^*^Statistically significant at *p* value < 0.05

Bold values indicate statistically significant differences

**Fig. 2:**
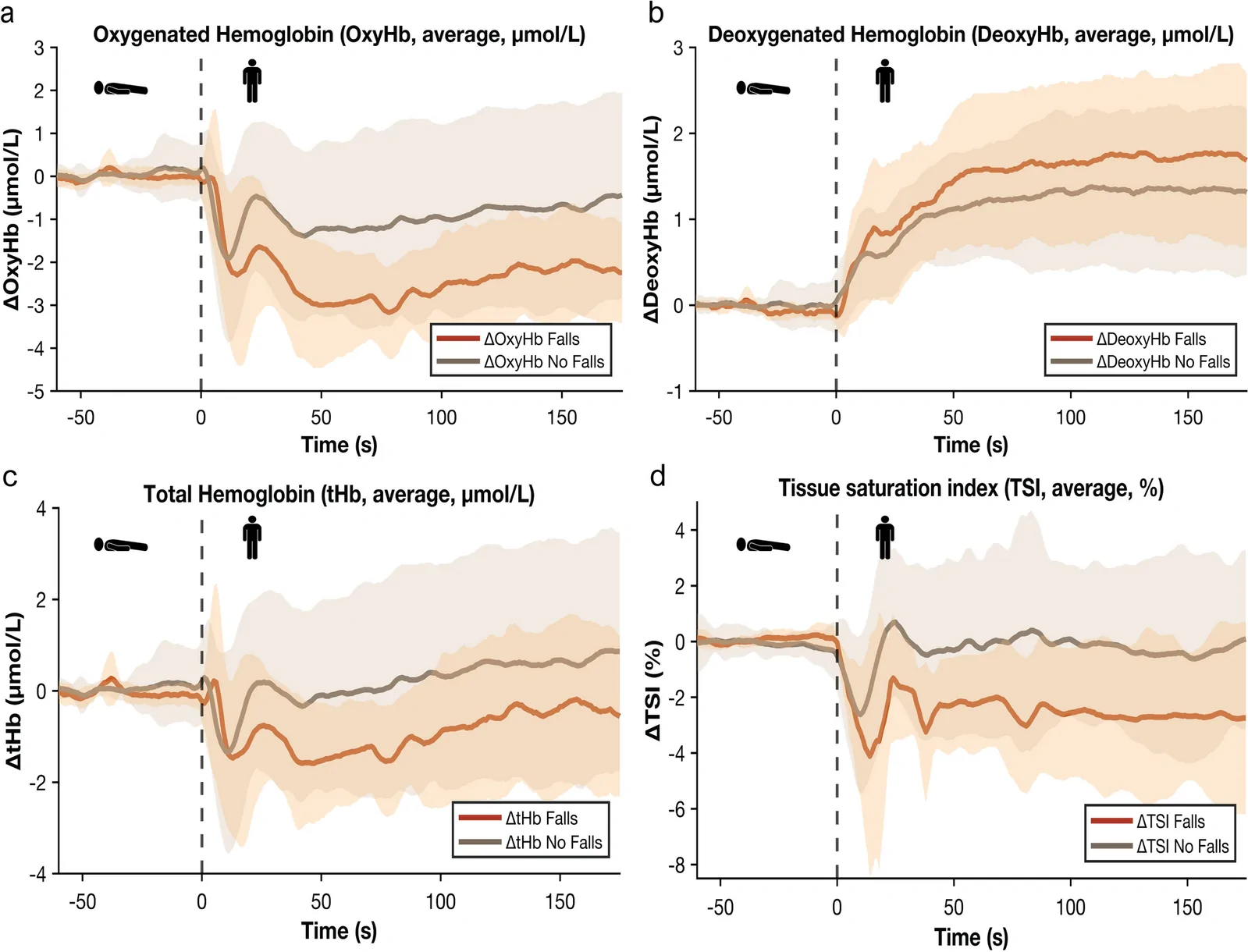
Orthostatic cerebral oxygenation responses for participants with or without positive falls history (supine-to-stand maneuver) for a. Oxygenated Hemoglobin; b. Deoxygenated Hemoglobin; c. Total Hemoglobin and d. Tissue Saturation Index, mean ± SD. Vertical black dashed line indicates moment of standing up

Participants with a history of falls exhibited distinct relative cerebral oxygenation responses during the supine-to-stand maneuver, showing more pronounced and sustained reductions in OxyHb, TSI, and tHb compared to those without a history of falls, without returning to baseline levels within 3 min of standing (Fig. 2). Statistically significant group differences were observed for several OxyHb parameters, including overshoot, nadir-to-overshoot amplitude, steady-state, and both initial and late recovery amplitudes (all *p* < 0.05; Table 2). TSI responses also differed significantly, with lower overshoot, steady-state, and initial recovery amplitude, as well as significantly lower TSI at 20 s and 40 s after standing among participants with a history of falls (all *p* < 0.05). In addition, the tHb nadir-to-overshoot amplitude was significantly smaller in participants with a history of falls (*p* = 0.009; Supplementary Table 1).

**Table 2:** Comparison of oxygenated hemoglobin and tissue saturation index responses upon standing between participants with and without a fall in the previous year (supine-to-stand maneuver; mean ± SD or median [IQR])

Variable^a^	Fall previous year	No fall previous year	*P* value*
	(*n* = 7)	(*n* = 22)	
*Oxygenated hemoglobin (OxyHb; μmol/L)*			
OxyHb nadir (within 30 s)	−2.6 ± 1.8	−2.1 ± 1.9	0.556
OxyHb overshoot (nadir time + 2 s to 60 s)	−1.7 ± 1.2	−0.1 ± 1.8	**0.018**
OxyHb nadir-to-overshoot	0.9 ± 0.8	2.0 ± 1.2	**0.012**
OxyHb recovery rate	0.1 ± 0.1	0.1 ± 0.1	0.224
OxyHb steady-state (60-120 s)	−2.7 ± 1.1	−1.0 ± 2.2	**0.012**
OxyHb initial recovery amplitude	−1.7 ± 1.2	−0.1 ± 1.8	**0.018**
OxyHb late recovery amplitude	−2.5 ± 1.1	−0.9 ± 2.3	**0.022**
OxyHb at 20 s	−1.9 ± 1.9	−0.6 ± 1.8	0.147
OxyHb at 30 s	−1.9 ± 1.1	−0.8 ± 1.9	0.074
OxyHb at 40 s	−2.7 ± 1.5	−1.3 ± 2.0	0.069
*Tissue saturation index (TSI; %)*			
TSI nadir (within 30 s)	−3.6 [−5.8, −1.5]	−2.5 [−3.8, −1.0]	0.460
TSI overshoot (nadir time + 2 s to 60 s)	−1.0 ± 2.2	1.4 ± 3.0	**0.038**
TSI nadir-to-overshoot	1.6 [0.6, 3.8]	3.5 [1.5, 5.5]	0.241
TSI recovery rate	0.2 [0, 0.3]	0.3 [0.1, 0.5]	0.231
TSI steady-state (60-120 s)	−2.5 ± 2.3	0.0 ± 3.2	**0.039**
TSI initial recovery amplitude	−1.0 ± 2.2	1.5 ± 3.0	**0.038**
TSI late recovery amplitude	−2.6 ± 2.5	−0.2 ± 3.2	0.058
TSI at 20 s	−3.1 [-4.2, -0.7]	0.2 [−0.1, 1.8]	**0.013**
TSI at 30 s	−1.7 ± 1.7	0.1 ± 2.7	0.059
TSI at 40 s	−3.0 [-3.8, -1.8]	−0.3 [−1.1, 0.6]	**0.012**

*IQR* interquartile range (25th to 75th percentile), *OxyHb* oxygenated hemoglobin, *s* second, *SD* standard deviation, *TSI* tissue saturation index

^**a**^Group differences between participants who experienced falls and those who did not were analyzed using an independent t-test for normally distributed continuous variables and a Mann–Whitney *U* test for non-normally distributed variables

^*^Statistically significant at *p* < 0.05; trends were considered at a *p* value of < 0.1 [25, 43]

Bold values indicate statistically significant differences

In contrast, no significant group differences were observed in DeoxyHb parameters. Likewise, no cerebral oxygenation response parameters (derived from OxyHb, DeoxyHb, tHb, TSI signals) differed significantly between participants with and without a history of falls during the sit-to-stand maneuver (Supplementary Table 2 and Supplementary Table 3).

In logistic regression analyses based on the supine-to-stand maneuver (Table 3), lower values across OxyHb and TSI parameters were associated with higher fall risk. After adjusting for age and sex, a lower OxyHb nadir-to-overshoot amplitude (within the first minute post-stand) was significantly associated with a positive history of falls (OR = 5.20, 95% CI 1.06–25.57, *p* = 0.042). Among TSI parameters, lower overshoot (OR = 1.86, 95% CI 1.04 -3.33, *p* = 0.038), lower initial recovery amplitude (OR = 1.86, 95% CI 1.04 −3.34, *p* = 0.037), and lower steady-state values from 60 to 120 s after standing (OR = 1.56, 95% CI 1.01–2.42, *p* = 0.044) were also significantly associated with a history of falls. Other parameters showed trends toward significance (all *p* < 0.1).

**Table 3:** Association between orthostatic cerebral oxygenation responses and history of falls (supine-to-stand maneuver)

	Unadjusted			Adjusted^c^		
Variable^a^	OR of falls history^b^	95%CI	*P* value*	OR of falls history^b^	95%CI	*P* value*
OxyHb overshoot (nadir time + 2 s to 60 s)	1.82	0.99–3.35	0.054	1.90	0.97–3.73	0.061
OxyHb nadir-to-overshoot	3.02	1.02–8.94	**0.046**	5.20	1.06–25.57	**0.042**
OxyHb steady-state (60-120 s)	1.64	0.94–2.87	0.080	1.80	0.94–3.46	0.077
OxyHb initial recovery amplitude	1.82	0.99–3.34	0.054	1.90	0.97–3.73	0.061
OxyHb late recovery amplitude	1.54	0.92–2.61	0.103	1.65	0.92–2.98	0.095
TSI overshoot (nadir time + 2 s to 60 s)	1.44	0.97–2.13	0.072	1.86	1.04–3.33	**0.038**
TSI steady-state (60-120 s)	1.37	0.96–1.94	0.082	1.56	1.01–2.42	**0.044**
TSI initial recovery amplitude	1.44	0.97–2.14	0.071	1.86	1.04–3.34	**0.037**
TSI at 20 s	1.28	0.99–1.67	0.060	1.28	0.98–1.68	0.072
TSI at 40 s	1.39	0.96–2.00	0.081	1.42	0.98–2.06	0.061
tHb nadir-to-overshoot	2.86	0.87–9.30	0.081	6.04	0.94–38.97	0.059

*CI* confidence interval, *OR* odds ratio, *OxyHb* oxygenated hemoglobin, *s* second, *TSI* tissue saturation index, *tHb* total hemoglobin

^**a**^Cerebral oxygenation response parameters that differed significantly between participants with and without a history of falls were entered into logistic regression models to explore their association with falls

^**b**^Parameters were multiplied by -1 prior to analysis to ensure OR > 1 reflects higher fall risk associated with lower original values

^**c**^Adjusted for age and sex

^*^Statistically significant at *p* < 0.05; trends were considered at a *p* value of < 0.1[25, 43]

Bold values indicate statistically significant associations

## Discussion

This study demonstrates that attenuated and delayed recovery of OxyHb and TSI during the first minutes after standing from a supine position are significantly associated with falls in older adults. These NIRS-derived parameters, reflecting impaired cerebral perfusion and sustained hypoperfusion, may therefore be useful for fall-risk assessment in older adults. In particular, lower OxyHb nadir-to-overshoot, TSI overshoot, and TSI initial recovery amplitude (all within the first minute), and reduced TSI steady-state values (60–120 s), emerged as key markers associated with falls.

The strongest group differences and associations with falls occurred within the first minute after standing. A lower OxyHb nadir-to-overshoot amplitude, indicating impaired recovery of cerebral oxygenation after the initial postural drop, was significantly associated with falls. This parameter captures the brain’s rapid cerebrovascular compensation to gravitationally induced hypoperfusion [44]. Slower recovery of cerebral oxygenation may reflect impaired cerebral autoregulatory responses and/or reduced autonomic cardiovascular compensation following postural change, limiting the ability to rapidly restore cerebral oxygenation after the initial orthostatic challenge [5, 44, 45]. Such impairments may indicate diminished homeostatic responses to orthostatic stress, baroreflex dysfunction, or broader frailty-related physiological vulnerability, which could contribute to increased fall risk [5, 44, 46]. Consistent with findings from syncope research, NIRS-detected significant reductions in OxyHb have been shown to precede both the onset of syncope symptoms and observable BP changes [13, 14, 23], highlighting the sensitivity of NIRS to early cerebral perfusion deficits. Previous work from our team in PROHEALTH cohort also found OxyHb responses at 30–40 s and 1-min recovery correlated with BP recovery, further reinforcing the physiological relevance of this early window [12].

Similarly, lower TSI overshoot and initial recovery amplitudes (within the first minute) were associated with falls, suggesting impaired cerebral perfusion recovery. These parameters also capture the brain’s ability to restore oxygenation after standing [44]. Our findings align with the TILDA cohort, where a > 1–2% TSI drop below baseline indicated cerebrovascular impairment [21]. In our study, TSI dropped by over 2% at 40 s among participants with a history of falls, a clinically relevant timepoint consistent with TILDA’s OH at 40 s (OH40), which has previously been linked to falls risk [2, 38]. Together, these findings highlight the diagnostic potential of early-phase (i.e., within the first minute after standing) NIRS dynamics. This is supported by prior studies showing that poor TSI recovery at 30 s was associated with greater multimorbidity in older adults, independent of peripheral hemodynamics [27], supporting the added value of NIRS beyond traditional hemodynamics. Additionally, primary NIRS changes were observed within the first 30 s in syncope patients, and impaired cerebral oxygenation recovery around 20–27 s was linked to frailty [28, 34]. In addition, a recent TILDA study found greater cerebral hypoperfusion in participants with OH at 30 and 60 s after standing and showed that combined OH and larger TSI drops were strongly associated with future falls [46].

Beyond early responses, reduced TSI steady-state values (60–120 s after standing) were also significantly associated with falls, indicating impaired sustained cerebral perfusion recovery. This period reflects the brain’s capacity to maintain cerebral perfusion after the initial orthostatic response. The steady-state parameter was included to detect prolonged hypoperfusion or impaired autoregulatory function. Our findings align with those from older adults with multimorbidity [27], and with TILDA Finometer findings linking sustained OH during this interval to unexplained and injurious falls [38]. In all, changes in NIRS-measured cerebral oxygenation during both the early and steady-state phases capture important aspects of cerebrovascular regulation and may help identify individuals at higher risk of orthostatic intolerance and falls.

In addition, supine-to-stand testing is more effective than sit-to-stand in detecting cerebral hypoperfusion and fall risk via NIRS. Multiple cerebral oxygenation parameters from the supine-to-stand maneuver were significantly associated with falls, whereas no such associations were observed with the sit-to-stand maneuver. This disparity likely reflects greater gravitational stress and venous pooling in supine-to-stand transitions, imposing a stronger challenge to cerebral perfusion and autoregulation [44, 47]. These findings are consistent with Mol et al. (2022), who advocated supine-to-stand as the preferred approach for assessing orthostatic cerebral oxygenation [10], and with Finometer studies showing larger BP drops and higher OH prevalence in supine-to-stand tests, further underscoring its clinical value in fall-risk assessment [12, 48].

Strengths of this exploratory study include the use of continuous bilateral NIRS monitoring and evaluation of multiple physiologically relevant parameters, offering a comprehensive assessment of cerebral oxygenation during postural changes. Few prior studies have linked cerebral oxygenation dynamics to fall risk in older adults, highlighting the potential clinical value of our findings. If validated in larger prospective studies, NIRS may complement standard orthostatic assessments by providing additional information on cerebral hypoperfusion and identifying older adults at increased risk of falls. Among the parameters examined, key markers include OxyHb and TSI parameters within the first minute after standing, along with TSI steady-state responses at 60–120 s. Specifically, TSI initial recovery amplitude appears particularly promising because it reflects early cerebral oxygenation recovery following standing and was significantly associated with falls in this study. The orthostatic protocol (supine-to-stand and sit-to-stand) was conducted under guided supervision and continuous monitoring, ensuring safety and standardized assessment of orthostatic cerebrovascular responses in relatively healthy older adults.

Limitations include a modest sample size and a low number of fall events, which may limit statistical power, increase the risk of overfitting in the adjusted logistic regression models, and reduce the robustness of the conclusions. In addition, the retrospective nature of the analysis limits the ability to predict incident falls. Therefore, the findings should be interpreted as exploratory and require validation in larger prospective cohorts. The relevance of this study is primarily as a framework for larger prospective studies, rather than as definitive evidence for clinical implementation. Additional limitations include limited covariate adjustment (only for age and sex), excluding factors such as cardiovascular comorbidities and medication, and reliance on self-reported falls, which may introduce recall bias or underreporting. Furthermore, this cohort comprised relatively healthy older adults, limiting the generalizability of the findings to more frail and heterogeneous populations.

Future studies should clarify the potential role of NIRS in more frail and heterogeneous older populations, including individuals with cognitive impairment and those with neurological disorders, and explore whether NIRS provides added value beyond comprehensive, standardized fall assessments incorporating established risk factors such as frailty, gait, and balance. Larger, prospective studies are needed to validate these findings, refine diagnostic thresholds, and determine whether cerebral oxygenation dynamics independently predict future falls. If confirmed, integrating NIRS into standard orthostatic assessments could enable earlier detection of cerebral hypoperfusion and OH-related fall risk, facilitating more targeted fall-prevention strategies in older adults.

## Conclusions

This study found that impaired OxyHb and TSI responses within the first minute after standing, as well as reduced TSI during the sustained recovery phase (60–120 s), were significantly associated with falls in older adults. Key NIRS-measured parameters, including OxyHb nadir-to-overshoot, TSI (overshoot, initial recovery amplitude, and steady-state values), emerged as promising markers of orthostatic hypoperfusion-related fall risk. Supine-to-stand testing appeared more sensitive than sit-to-stand for detecting these features.

Overall, these findings support the clinical potential of NIRS as a practical, non-invasive tool for assessing fall risk related to orthostatic intolerance and orthostatic cerebral hypoperfusion. Large-scale prospective studies are required to validate these results and establish diagnostic thresholds for clinical implementation.

## Supplementary Information

Below is the link to the electronic supplementary material.

Supplementary file1 (DOCX 124 KB)

## Data Availability

In compliance with European data protection laws and the conditions of the trial's ethical approval, we are unable to disclose any patients’ personal data, even if anonymized. Researchers interested in access to the cohort data may upon request apply for access.
