# Supplementary material for: Cerebral oxygenation response to postural change using continuous near-infrared spectroscopy (NIRS) and its association with falls in older adults
Source: Eur Geriatr Med. 2026 Jun 29;17(4):2301–11. doi: 10.1007/s41999-026-01538-3 (PMC13562277; doi:10.1007/s41999-026-01538-3)
Supplement: Supplementary file 1 [file 41999_2026_1538_MOESM1_ESM.docx]

**Supplementary**

**Cerebral oxygenation response to postural change using continuous near-infrared spectroscopy (NIRS) and its association with falls in older adults**

**Supplementary Table 1** Comparison of deoxygenated hemoglobin and total hemoglobin responses upon standing between participants with or without a fall in the previous year (supine-to-stand maneuver; mean±SD or median [IQR])

| **Variable^a^** | **Fall previous year** | **No fall previous year** | **P-value*** |
| --- | --- | --- | --- |
|  | (n=7) | (n=22) |  |
| Deoxygenated hemoglobin (DeoxyHb; μmol/L) |  |  |  |
| DeoxyHb peak (within 30s) | 1.2 ± 0.7 | 1.0 ± 0.5 | 0.495 |
| DeoxyHb steady-state (60-120s) | 1.6 ± 0.9 | 1.3 ± 0.8 | 0.382 |
| DeoxyHb initial recovery amplitude | 0.8 ± 0.9 | 0.7 ± 0.5 | 0.679 |
| DeoxyHb late recovery amplitude | 1.7 ± 0.9 | 1.3 ± 0.9 | 0.362 |
| DeoxyHb at 20s | 0.8 ± 0.8 | 0.6 ± 0.5 | 0.457 |
| DeoxyHb at 30s | 1.2 [0.4, 1.7] | 0.9 [0.5, 1.4] | 0.505 |
| DeoxyHb at 40s | 1.2 ± 0.7 | 1.0 ± 0.6 | 0.628 |
| Total hemoglobin (tHb; μmol/L) |  |  |  |
| tHb nadir (within 30s) | -1.8 ± 1.5 | -1.6 ± 2.1 | 0.765 |
| tHb overshoot (nadir time+2s to 60s) | -0.4 ± 1.5 | 0.8 ± 1.9 | 0.104 |
| tHb nadir-to-overshoot | 1.4 ± 0.5 | 2.4 ± 1.4 | **0.009** |
| tHb steady-state (60-120s) | -1.1 ± 1.3 | 0.3 ± 2.4 | 0.075 |
| tHb initial recovery amplitude | -0.4 ± 1.5 | 0.8 ± 1.9 | 0.105 |
| tHb late recovery amplitude | -0.8 ± 1.5 | 0.4 ± 2.5 | 0.143 |
| tHb at 20s | -1.1 ± 1.9 | 0.0 ± 2.0 | 0.227 |
| tHb at 30s | -0.8 ± 1.1 | 0.1 ± 2.1 | 0.139 |
| tHb at 40s | -1.5 ± 1.5 | -0.3 ± 2.1 | 0.106 |

DeoxyHb, deoxygenated hemoglobin; IQR, interquartile range (25th to 75th percentile); s, second; SD, standard deviation; tHb, total hemoglobin.

**^a^** Group differences between participants who experienced falls and those who did not were analyzed using an independent t-test for normally distributed continuous variables and a Mann-Whitney U test for non-normally distributed variables.

* Statistically significant differences (*p*<0.05) are shown in bold; trends were considered at a p-value of <0.1.

**Supplementary Table 2** Comparison of cerebral oxygenation responses upon standing between participants with or without a fall in the previous year (sit-to-stand maneuver; mean±SD or median [IQR])

| **Variable^a^** | **Fall previous year** | **No fall previous year** | ***P*-value*** |
| --- | --- | --- | --- |
|  | (n=7) | (n=23) |  |
| Oxygenated Hemoglobin (OxyHb; μmol/L) |  |  |  |
| OxyHb nadir (within 30s) | -1.7 [-1.9, -0.9] | -1.2 [-2.1, -0.9] | 0.806 |
| OxyHb overshoot (nadir time+2s to 60s) | -0.3 [-0.6, 0.7] | -0.3 [-0.6, 0.1] | 0.863 |
| OxyHb nadir-to-overshoot | 1.4 ± 0.7 | 1.3 ± 0.8 | 0.742 |
| OxyHb recovery rate | 0.07 [0.1, 0.1] | 0.07 [0.0, 0.1] | 0.980 |
| OxyHb steady-state (60-120s) | -1.3 [-1.5, -0.4] | -0.7 [-1.1, -0.5] | 0.315 |
| OxyHb initial recovery amplitude | -0.3 [-0.6, 0.6] | -0.3 [-0.5, 0.1] | 0.825 |
| OxyHb late recovery amplitude | -1.2 [-1.5, -0.3] | -0.7 [-1.1, -0.5] | 0.292 |
| OxyHb at 20s | -0.5 ± 1.6 | -0.4 ± 0.5 | 0.882 |
| OxyHb at 30s | -0.7 [-1.1, -0.3] | -0.8 [-1.2, -0.4] | 0.731 |
| OxyHb at 40s | -1.2 [-1.2, -0.8] | -0.7 [-1.2, -0.4] | 0.403 |
| Deoxygenated hemoglobin (DeoxyHb; μmol/L) |  |  |  |
| DeoxyHb peak (within 30s) | 0.4 ± 0.5 | 0.2 ± 0.3 | 0.537 |
| DeoxyHb steady-state (60-120s) | 0.3 ± 0.6 | 0.2 ± 0.3 | 0.473 |
| DeoxyHb initial recovery amplitude | -0.2 [-0.4, 0.1] | -0.2 [-0.3, 0.1] | 0.902 |
| DeoxyHb late recovery amplitude | 0.3 ± 0.7 | 0.2 ± 0.3 | 0.517 |
| DeoxyHb at 20s | 0.2 ± 0.6 | 0.0 ± 0.4 | 0.440 |
| DeoxyHb at 30s | 0.1 ± 0.5 | 0.0 ± 0.3 | 0.439 |
| DeoxyHb at 40s | 0.1 ± 0.4 | 0.1 ± 0.4 | 0.832 |
| Total hemoglobin (tHb; μmol/L) |  |  |  |
| tHb nadir (within 30s) | -1.7 [-1.9, -0.8] | -1.3 [-2.2, -1.0] | 0.961 |
| tHb overshoot (nadir time+2s to 60s) | -0.3 [-0.8, 1.4] | -0.2 [-0.5, 0.1] | 0.902 |
| tHb nadir-to-overshoot | 1.7 ± 0.9 | 1.5 ± 1.0 | 0.643 |
| tHb recovery rate | 0.08 [0.1, 0.1] | 0.08 [0.0, 0.1] | 0.659 |
| tHb steady-state (60-120s) | -1.1 [-1.4, 0.3] | -0.6 [-0.9, -0.3] | 0.641 |
| tHb initial recovery amplitude | -0.3 [-0.8, 1.4] | -0.2 [-0.5, 0.1] | 1.000 |
| tHb late recovery amplitude | -0.9 [-1.4, 0.4] | -0.6 [-0.8, -0.4] | 0.677 |
| tHb at 20s | -0.3 ± 2.1 | -0.4 ± 0.7 | 0.874 |
| tHb at 30s | -0.9 [-1.2, 0.0] | -0.6 [-1.3, -0.4] | 0.787 |
| tHb at 40s | -0.8 ± 1.5 | -0.7 ± 0.9 | 0.897 |

DeoxyHb, deoxygenated hemoglobin; IQR, interquartile range (25th to 75th percentile); OxyHb, oxygenated hemoglobin; s, second; SD, standard deviation; tHb, total hemoglobin.

**^a^** Group differences between participants who experienced falls and those who did not were analyzed using an independent t-test for normally distributed continuous variables and a Mann-Whitney U test for non-normally distributed variables.

* Statistically significant differences were considered at a p-value of <0.05.

**Supplementary Table 3** Comparison of tissue saturation index responses upon standing between participants with or without a fall in the previous year (sit-to-stand maneuver; mean±SD or median [IQR])

| **Variable^a^** | **Fall previous year** | **No fall previous year** | ***P*-value*** |
| --- | --- | --- | --- |
|  | (n=6) | (n=23) |  |
| Tissue saturation index (TSI; %) |  |  |  |
| TSI nadir (within 30s) | -0.6 [-0.6, -0.4] | -0.8 [-1.2, -0.3] | 0.725 |
| TSI overshoot (nadir time+2s to 60s) | -0.1 [-0.2, 0.2] | 0.0 [-0.2, 0.2] | 0.745 |
| TSI nadir-to-overshoot | 0.5 [0.3, 1.1] | 0.7 [0.3, 1.3] | 0.936 |
| TSI steady-state (60-120s) | -0.5 [-0.7, -0.2] | -0.2 [-0.6, 0.0] | 0.282 |
| TSI initial recovery amplitude | -0.1 [-0.2, 0.1] | 0.0 [-0.2, 0.3] | 0.706 |
| TSI late recovery amplitude | -0.4 [-0.7, -0.2] | -0.2 [-0.7, 0.0] | 0.518 |
| TSI at 20s | -0.2 [-0.4, -0.2] | 0.0 [-0.2, 0.1] | 0.083 |
| TSI at 30s | -0.3 [-0.5, -0.3] | -0.3 [-0.8, 0.0] | 0.892 |
| TSI at 40s | -0.4 [-0.6, -0.2] | -0.1 [-0.8, 0.1] | 0.626 |

IQR, interquartile range (25th to 75th percentile); s, second; SD, standard deviation; TSI, tissue saturation index.

**^a^** Group differences between participants who experienced falls and those who did not were analyzed using an independent t-test for normally distributed continuous variables and a Mann-Whitney U test for non-normally distributed variables.

* Statistically significant differences were considered at a p-value of <0.05; trends were considered at a p-value of <0.1.
